# Supplementary material for: Transcriptome Analysis Reveals Vimentin-Induced Disruption of Cell–Cell Associations Augments Breast Cancer Cell Migration
Source: Cells. 2022 Dec 13;11(24):4035. doi: 10.3390/cells11244035 (PMC9776984; doi:10.3390/cells11244035)
Supplement: Supplementary file 1 [file cells-11-04035-s001.zip › DEGs and enrichment analysis.zip!/DEGs and enrichment analysis/WT_VIMvsVector KEGG/DOWN/WT_VIMvsVector_down.bar.pdf]

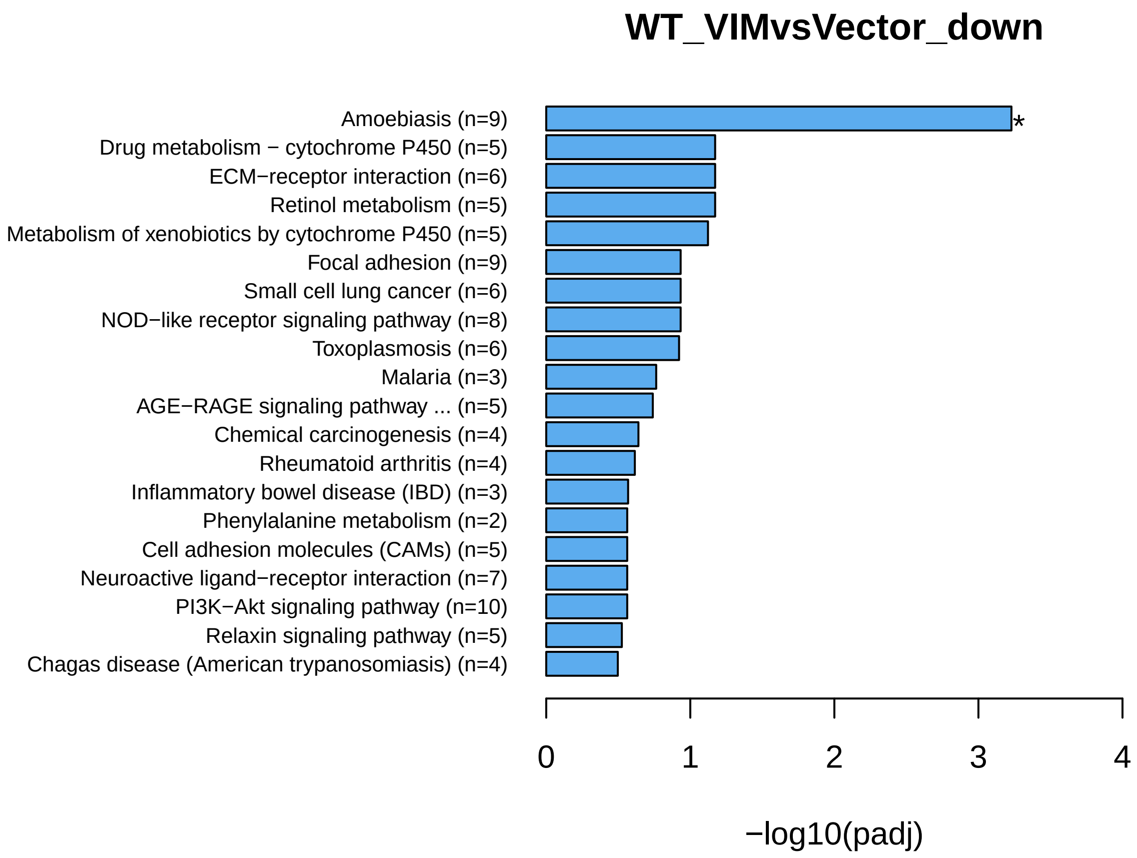
WT_VIMvsVector_down
Amoebiasis (n=9)
Drug metabolism − cytochrome P450 (n=5)
ECM−receptor interaction (n=6)
Retinol metabolism (n=5)
Metabolism of xenobiotics by cytochrome P450 (n=5)
Focal adhesion (n=9)
Small cell lung cancer (n=6)
NOD−like receptor signaling pathway (n=8)
Toxoplasmosis (n=6)
Malaria (n=3)
AGE−RAGE signaling pathway ... (n=5)
Chemical carcinogenesis (n=4)
Rheumatoid arthritis (n=4)
Inflammatory bowel disease (IBD) (n=3)
Phenylalanine metabolism (n=2)
Cell adhesion molecules (CAMs) (n=5)
Neuroactive ligand−receptor interaction (n=7)
PI3K−Akt signaling pathway (n=10)
Relaxin signaling pathway (n=5)
Chagas disease (American trypanosomiasis) (n=4)
*
0
1
2
3
4
−log10(padj)
